# Therapeutic Futility in Nursing: A Focus Group

**DOI:** 10.1177/23779608221134768

**Published:** 2022-10-31

**Authors:** João V. Vieira, Sérgio Deodato, Felismina Mendes

**Affiliations:** 1 Centre for Interdisciplinary Health Research, 59207 Universidade Católica Portuguesa, Lisboa, Portugal; 2 Department of Health, Instituto Politécnico de Beja, Beja, Portugal; 3 Comprehensive Health Research Centre, 70989 Universidade de Évora, Évora, Portugal

**Keywords:** critical care nursing, ethics, futility, intensive care units, nursing

## Abstract

**Introduction:** The implementation of futile nursing interventions seems to be a persistent problem in adult intensive care units. Understanding this phenomenon can contribute to its prevention and all deleterious effects associated with it. **Objective:** To identify the perceptions of expert nurses from adult intensive care units about therapeutic futility in nursing. **Methods:** This study consists of a conventional content analysis. Data was collected through a focus group interview that included five expert nurses in adult intensive care, with a minimum of fifteen years of professional experience in intensive care. To analyze the information, the technique of thematic categorical analysis was used, according to Bardin. **Results:** Four central categories were identified for the topic under study, for which several subcategories were identified that allow a better understanding of this phenomenon. **Conclusion:** Adult intensive care expert nurses advocate that therapeutic futility in nursing is a reality perceived by teams and families, which should be avoided due to the risk of potentiating the implementation of ethically reprehensible care.

## Introduction

Intensive care units are highly advanced and organized systems dedicated to providing care to critically ill patients. Thanks to a wide monitoring capacity and multiple modalities of physiological organ support, these units enable the provision of care to people with highly complex critical illness. These units are equipped with a multidisciplinary team that is systematically dedicated to the care of people who have developed, or are at risk of developing, acute organic dysfunction that puts their lives at risk. However, despite the noble availability of resources, there are situations of high vulnerability and irreversibility that are impossible to reverse even in these environments (Vieira et al., 2021a).

Therapeutic futility has been studied for a long time, with evidence from records dating to the time of Hippocrates. However, the term futile care only emerged in the health literature in the 80s of the last century (McCord, 2017). Since then, despite numerous efforts to define therapeutic futility, it continues to be seen that there is no universal acceptance of this concept, although some central elements of it are identified when futility refers to health in general, namely, the diagnosis of futility is closely related to clinical judgment, futility has both quantitative and qualitative roots, futility is always appreciated a posteriori, futility is related to the lack of benefit (Vieira et al., 2021b).

## Review of the Literature

In general, nurses are healthcare professionals who demonstrate a high level of moral sensitivity to ethical issues in clinical practice (Luca et al., 2021). Intensive care nurses consider that therapeutic futility is a recurring problem in their practice and that the perception of futile care has a direct negative relationship with their behavior when providing nursing care (Vieira et al., 2021c; Rostami et al., 2019). In these environments, this conception often generates debate and conflict, especially among nurses (Pishgooie et al., 2019), and its contribution to the reduction of job satisfaction and the exhaustion of these professionals has been proven Ozden et al., 2013).

Although it is consensual that therapeutic futility occurs frequently, especially in certain environments that are characterized by the existence of technological and human resources that make it possible to prolong life, such as intensive care units, this practice continues to be verified frequently through the implementation of inappropriate interventions that do not contribute to the improvement of the prognosis or to the relief of symptoms, aiming exclusively at prolonging life (Mohammed & Peter, 2009).

The underlying reasons for therapeutic futility can be varied, but in general they fall into three main categories, such as patients and family members request for continuing life-sustaining treatments, personal reasons of health professionals, cultural and organizational structure (Aghabarary & Nayeri, 2017). Some authors even mention that the main reason for the implementation of futile nursing interventions is associated with patient/family requests. In these circumstances, the nurse must act as a mediator and seek to resolve any differences between the patient/family and health professionals. However, for this mediation to be carried out with quality, it is important to understand under what circumstances these interventions are promoted (Kadooka et al., 2014). The lack of an ecumenically accepted concept of therapeutic futility in nursing may be one of the reasons why, in intensive care units, it can perpetuate a culture of denial of death, in which nurses contribute to the provision of care that may exceed ethically reprehensible limits. For this reason, regardless of the inherent complexity of this concept, it is urgent to promote a discussion that makes it possible to identify and define it.

Therapeutic futility has been subject of discussion all over the world. However, despite several studies carried out by nurses, there is still a lack of consensus regarding the definition and early identification of therapeutic futility, which contributes to the persistence of this phenomenon. In Portugal, the debate on the topic of therapeutic futility has become more evident in the last decade, which has contributed to the increase in scientific publications by national authors, especially in the areas of intensive care (Teixeira et al., 2012; Vieira et al., 2021c) and palliative care (Domingues et al., 2015; Graça et al., 2021; Marinho & Casanova, 2019). Most studies are unanimous in pointing to the persistence of situations of therapeutic futility, especially in end-of-life situations, associated with several factors, such as communication problems, discrepancies in the judgment of health professionals, insufficiencies in training/education to deal with complex situations and problems associated with therapy.

## Objective

To identify the perceptions of expert nurses from adult intensive care units about therapeutic futility in nursing.

## Method

### Design

Focus group interview conducted in November 2021, by videoconference, moderated by the principal investigator and supervised by an ethical decision specialist. A conventional content analysis was carried out through a thematic categorical analysis. This approach allowed the direct identification of categories extracted from the data collection. The study occurred between August 2021 and July 2022.

Data were collected using a focus group interview, carried out to stimulate participation and to enhance the quantity and quality of the narratives. This focus group interview, carried out in November 2021, lasted 127 min and included the following questions: (1) Tell us about a situation in which you think you provided nursing care (or attended the provision of nursing care) more than what was necessary for the specific person; (2) What did you do in that situation, what did you consider to have been futile or too much, that is, beyond what was necessary? (3) Why did you consider/think that what you did was too much? Was it just your perception, or was it a generalized appreciation? (4) When you think of therapeutic futility in nursing, what terminology/terms do you think of?

The focus group interview was carried out using videoconferencing. Despite that the most used strategy for this method is still face-to-face, recent studies point to benefits associated with videoconferencing, namely, the possibility of bringing together participants from significantly dispersed geographical areas and the ease of proceeding with the full recording of all discussions provided, provided that with the express authorization of all participants (Matthews et al., 2018).

### Research Question

What are the perceptions of expert nurses from adult intensive care units about therapeutic futility in nursing.

### Sample/Inclusion Criteria

For this study, a non-probabilistic sampling method was used, specifically, convenience sampling. Study participants were intentionally selected and consisted exclusively of nurses who are experts in adult intensive care. The intention of the sample for a study of a qualitative nature is justified by the selection of expert participants whose anticipated richness and depth of contribution could not be neglected. Inclusion criteria were: (1) adult intensive care nurses; (2) nurses with a minimum of fifteen years of professional experience in an adult intensive care unit; (3) advanced knowledge of the topic under study and interest in participating.

A total of five nurses (Table 1) participated in the study, three men and two women. The nurses’ age ranged between 40 years and 66 years (mean = 54.2; median = 54; standard deviation = 9.6). The length of professional experience ranged from 18 years to 43 years (mean = 30.4 years; median = 29; standard deviation = 9.12). The length of experience in the adult intensive care units ranged from 18 years to 34 years (mean = 23.2; median = 20; standard deviation = 7.98).

**Table 1.** Characteristics of Focus Group Participants.

	Sex	Age	Length of professional experience	Length of experience in adult intensive care units	Academic degree	Professional category
P.1	M	54	29	29	MSc	Clinical Nurse Specialist
P.2	F	52	28	15	MSc	Clinical Nurse Specialist
P.3	F	66	43	20	MSc	Nurse Manager
P.4	M	56	34	34	PhD	Nurse Manager
P.5	M	40	18	18	MSc	Clinical Nurse Specialist

### Institutional Review Board Approval and Informed Consent

The study was approved by the Scientific Committee and the Ethics Committee of a University in Portugal (148_CES-UCP). Before conducting the focus group interview, confidentiality and anonymization were guaranteed and all participants in the focus group interview consented to their authorization. In addition, all participants were informed about all characteristics of the study, objectives, and the reasons behind the study. Voluntary participation was emphasized and during the focus group interview, anticipating that the narrative of delicate and complex situations could imply some negative emotion in the participants, a specialist in ethical decision was present for the eventual need to provide assistance in the field of moral suffering.

### Statistical Analysis

The analysis of the data collected was carried out between November 2021 and March 2022, in a first phase independently by two authors, after transcription of the audio to text. For the qualitative analysis of the information obtained, the content analysis technique was used, an information treatment technique commonly used in empirical research, particularly thematic categorical analysis (Bardin, 2016), with the purpose of obtaining, through systematic and objective procedures for the description of the content of messages, indicators that allow the inference of knowledge related to the opinions of the experts interviewed (Polit & Beck, 2017; Polit & Beck, 2018).

The operations to be carried out during the content analysis included the definition of categories, the definition of the units of analysis, and the quantification centered on the recording units of each indicator. The text, once transcribed, was analyzed using the NVivo® software.

Each of the authors listened and analyzed the transcript several times, to extract the categories identified according to the subcategories that emerged. All similar subcategories were classified into the same category and significantly similar categories were merged. Subsequently, the authors compared their results and whenever differences were found in the analysis, a discussion was promoted with the third author until a final consensus was reached. This analysis was performed exclusively by the authors. It should be noted that, both during the data collection phase and in the data analysis phase, the researchers were able to identify and keep in abeyance their convictions, beliefs, and opinions about the phenomenon under study, excluding all prepositions and convictions to confront the narratives obtained in the purest form (Polit & Beck, 2017; Polit & Beck, 2018).

To confirm the credibility of the data obtained, the principal investigator of this study established several contacts with the participants. Additionally, exhaustive research was carried out on the subject and a constant evaluation was promoted by experts on the subject under study. After transcription, the focus group interview was returned to all participants to confirm a true transcript. After identifying the codes, categories, and subcategories extracted, to confirm the reliability an evaluation was carried out by two experts.

## Results

The content analysis performed on the content extracted from the focus group interview made it possible to identify four general categories related to the topic under study of therapeutic futility in nursing, specifically, the situation of declared futility in nursing, futile nursing interventions, the recognition of therapeutic futility in nursing and the scope of therapeutic futility in nursing (Table 2). For each of the categories, several subcategories were identified.

**Table 2.** Categories and Subcategories That Emerged from the Content Analysis.

Category	Subcategory
Situation of declared futility in nursing	Biophysiological indicators incompatible with life
	Intensive care culture of life extension
	Surgical situations of high irreversibility, uncontrollable, associated with severe comorbidities
	Contexts in which, according to scientific evidence, the results are unattainable and do not justify the implementation of interventions
Futile nursing interventions	Interdependent nursing interventions
	Autonomous nursing interventions
	Interventions implemented exclusively by norms or protocols, routines, scores
	Interventions associated with exclusively diagnostic complementary exams
Recognition of therapeutic futility in nursing	By the nursing team
	By the family
Scope of therapeutic futility in nursing	Ridicule of care
	Transposing the limits of interventions and care
	No benefit
	Therapeutic incarceration

### Situation of Declared Therapeutic Futility in Nursing

In this category, four subcategories were identified in which nurses who are experts in adult intensive care recognize the existence of therapeutic futility in nursing in adult intensive care units. Situations in which the implementation of interventions is maintained despite evidence of biophysiological indicators incompatible with life: “*Patients who arrive in refractory septic shock*…” (P.1); “*Patients who essentially have a pH lower than seven…and in addition they have lactates that sometimes go as high as 15 and 20 mmol/ L*.” (P.1); “*People with severe, refractory hypotension, despite unreasonable administrations of vasoactive amines.*” (P.2). Persistence of care that results directly from the culture of intensive care that only aims to prolong life: “*We end up prolonging life in some situations*…” (P.4); “*We intensivists are a little accused of taking our attitude of interventionists.*" (P.4); "*…Use of some abused drugs to prolong this state of life…we nurses know, we clinicians know that we will not be able to have the quality of life in that person, we will just prolong it life.*” (P.4); “*The margin in which we have to intervene is very short and we only prolong this life a little*…” (P.4). People with surgical situations of high irreversibility associated with severe comorbidities: “*They are essentially surgical situations, often linked to comorbidities and that we quickly realize that they are situations that are not controllable*.” (P.3); “*Surgical situations of great complexity and gravity, which we quickly realize are irreversible*.” (P.3). Contexts in which, according to scientific evidence, results are unattainable and do not justify the implementation of interventions: “*Situations … with everything that has no positive purpose for the patient we are caring for*.” (P.5).

### Futile Nursing Interventions

There was almost general consensus in the category in which the participants identified the futile nursing interventions that they most frequently perceive, from which four subcategories stand out: participation in some interdependent nursing interventions, that are prescribed by other health professionals and in which the nurse assumes responsibility for their implementation, with particular emphasis on the placement of invasive clinical devices; autonomous nursing interventions, in which the nurse assumes responsibility for its prescription and implementation; interventions implemented exclusively by norms and protocols, routines or scores; the provision of care in complementary diagnostic exams that aim exclusively at a diagnosis and that do not offer any contribution to the improvement of the prognosis/outcome.

Regarding interdependent nursing interventions, some of the nurses’ statements included: “*The most penetrating ones and the ones that leave a lot of reservations are not the independent interventions, they are the interdependent ones*.” (P.3); “*The placement or change of catheters, central catheters, arterial lines, and that sometimes we think what is this, what is this for? Because putting a catheter in is highly futile in a patient, we know is going to die*.” (P.1). With regard to autonomous nursing interventions, despite some initial controversy among some participants of the focus group, regarding some specific nursing care with a direct relationship with basic needs, some testimonies emerged that gathered consensus: “*Some independent interventions /autonomous, in certain contexts, can be futile: positioning a person who is already in the process of end-of-life, unconscious, without any evidence in terms of monitoring that shows suffering, is futile for me*.” (P.5); “*Placing nasogastric tubes, if it is exclusive to prolong life, is futile. Hydrating a person to prolong life when the outcome is pointless is futile*.” (P.5).

In the subcategory of interventions implemented by preestablished routines, by norms or protocols, by scores, there were several statements in which it was possible to identify different situations that fall into this subcategory, such as: “*Interventions that are extremely futile are changes daily, in terminally ill patients, of catheters, probes… This makes no sense, including the monitoring of vital signs*.” (P.1); “…*routine positioning*." (P.1); “*Defensive nursing…it is prescribed, and the nurse has to do it*!” (P.2); “…*decubitus change because it is time to do it, regardless of the inexistence of benefit*." (P.2); “*Interventions because there is a score to meet… And scores can be used and manipulated*…” (P.3).

The last subcategory identified in this topic proved to be the one with the most registration units, with a broad consensus among all nurses who considered that nursing interventions associated with complementary diagnostic exams that aim exclusively at a diagnosis, when the inevitability of death is confirmed, are futile interventions: “*What bothers me most are the excessive diagnostic aids that we provide to patients when they don't need them for anything*.” (P.4); “…*which are of no use, for absolutely nothing, because they do not alter the decision-making process in any way*.” (P.4); “*we use the nurses’ work a lot when carrying out additional diagnostic exams that I think are not an asset for decision making and benefit*…” (P.4); “*More and more people in their intensive care unit use and abuse complementary exams that are exclusively diagnostic, increasingly differentiated, and the futility of these diagnostic means of diagnosis is something that afflicts me*.” (P.3).

### Recognition of Therapeutic Futility in Nursing

From the participants’ narratives, it is possible to identify two subcategories that refer to the recognition of therapeutic futility in nursing, namely, recognition by the nursing team and recognition by family members. Some testimonies of the participants include, regarding the recognition by the nurses: “*Our conversation, isn't it, our shift changes, our daily reflections*.” (P.4); “*for the sharing of opinions among the group*." (P.4); “*our experience that sometimes gives us, or rather, the experience is something that we bring from the fact that we have been working in the units for many years and we identify similar situations, and we see and know that the future of that person will be very identical to countless cases that have already passed us.*” (P.1).

With regard to the family's recognition of futility, it was a consideration presented by the participants that deserved significant emphasis, with several recording units identified in testimonies such as: “*The family itself says: look, isn't it time to stop? Sometimes it is at this point that the team clicks that we are actually making too many approaches*.” (P.1); “*Sometimes it is the family itself that tells us: I do not want it anymore, it's not worth it*.” (P.1); “*The family itself says: I don't want it anymore, don't make my father or mother suffer anymore*.” (P.1); “*It is the family member who says: look, maybe it's been too much, is not it, and I do not want to cause pain, I don't want to cause my father suffering*.” (P.2); “*They see that the time that passes, the person in the unit, and that there is no improvement in them and the fact that we are improving treatment and diagnosis techniques makes the family itself aware*.” (P.2); “*Don't you think it is good to stop because my father is suffering? And I am even a little astonished and wondering if that person is okay, but maybe he's correct*.” (P.1). This assessment of family members is attributed, by some participants, to the increase in health literacy, to which, according to nurses, the enormous ease of obtaining information contributes greatly.

### Scope of Therapeutic Futility in Nursing

Initially, some difficulty was recognized on the part of the participants in identifying elements to characterize the extent and scope of therapeutic futility in nursing, including some reservations when nurses were asked to present suggestions for terms/associated terminology to this conception. From the responses presented by the participants, four subcategories are recognized, particularly, ridicule of care, transposition of the limits of intervention and care, lack of benefit, and therapeutic incarceration.

In the first subcategory that was identified, ridicule of care, statements such as: “*When we talk about futility, I talk about ridicule, ridiculous things*." (P.4); “*It is something that is ridiculous*." (P.4); “…*it's ridiculous that we're doing something like that because it's unthinkable*…” (P.4); “*Look how ridiculous the situation is!” (P.4). Regarding the transposition of the limits of intervention and care, the second subcategory identified the expression “to exceed the limit*.” (P.2, P.3, P.5). Concerning the third subcategory, which refers to the lack of benefit, several registration units were identified, with emphasis on the following statement: “*It is everything that goes beyond the benefit for the patient*.” (P.2). Finally, on the last subcategory, which was called therapeutic incarceration, there were several statements made by nurses who are experts in adult intensive care, which include: “*For me, futility is incarceration*.” (P.3); “… *futility is therapeutic incarceration, it is a hopeless investment*.” (P.3); “*continue to make a series of interventions to the patient, knowing that the end is in sight*.” (P.3); “…*systematic surgeries… vasopressors in doses incompatible with life… continuous dialysis techniques knowing that the end is in sight, all this, and more, are therapeutic incarceration.*” (P.2).

On this issue of the scope of therapeutic futility in nursing, it is important to note that one of the participants mentioned the difficulty in identifying the limit of their performance in a personal situation, saying: “*Foreseeing that this could happen and thinking it was futile and that I would never do it, but when the time came I did it all because it was my mother*.” (P.3). On the contrary, in a situation that also involved a family member, another participant revealed a remarkable ability, in a situation of declared nursing therapeutic futility, not to persist in the implementation of interventions, declaring: “*I knew it was futile, that it was not over there… I do not, I didn't make my brother's comfort worse, and I know it. However, above all, I gave comfort to the family*.” (P.2).

## Discussion

The realization of this study made it possible to identify some of the perceptions that underlie and/or dominate the daily action that nurses in adult intensive care units have about therapeutic futility in nursing. The narratives of the participants, all experts in adult intensive care, with a minimum time of professional experience in these contexts of care practice of fifteen years, allowed the identification of four situations in which it is possible to recognize therapeutic futility in nursing, namely: situations in which the implementation of interventions is maintained despite the evidence of biophysiological indicators incompatible with life; persistence of care that results directly from the culture of intensive care that only aims at prolonging life; people with highly irreversible surgical situations associated with severe comorbidities; contexts in which, in the light of science and scientific evidence, results are unattainable and do not justify the implementation of interventions. Nurses confirmed, with consensus, that these are the four situations in which they have already recognized situations of therapeutic futility in nursing in the context of an adult intensive care unit.

In the category of futile nursing interventions, it was possible to recognize the existence of four subcategories, interdependent nursing interventions, autonomous nursing interventions, interventions implemented exclusively by norms or protocols, routines or scores, interventions associated with exclusively diagnostic complementary exams. It was with some surprise that one of the subcategories that the experts identified in this category was recognized, specifically, the subcategory of autonomous nursing interventions. It was anticipated that interdependent nursing interventions would be identified almost exclusively, that is, interventions that are prescribed by other professionals, namely, by the doctors, but that are implemented by the nurse, who assumes responsibility only for its implementation, highlighting the participation of invasive techniques, in the administration of therapy, among others. Notwithstanding the fact that the identification of autonomous nursing interventions was unexpected, the participants justified their acknowledgment stating that they are futile autonomous nursing interventions that are implemented when death is irreversible, presenting as example the placement of some clinical devices, such as nasogastric tubes exclusively to feed/hydrate the person, or the replacement of some devices such as peripheral catheters. This point generated a heated controversy, with three nurses vehemently speaking out against the argument presented regarding the therapeutic futility of food/hydration, regardless of the circumstances. Although some clinical objectives were not consensual among the participants, it was clear that in some circumstances there are autonomous nursing interventions that can be futile, such as the respective placement/replacement of clinical devices. Participants also mentioned that nursing interventions associated with complementary exams are exclusively diagnostic, that is, they do not contribute to decision making, and interventions and care provided exclusively by virtue of norms or protocols, routines, or scores, can be futile, reinforcing the importance of a personalized, regular, and systematic assessment.

The recognition of therapeutic futility in nursing, and inevitably of futile nursing interventions, was an aspect that gathered consensus among all participants, who identified that situations of therapeutic futility in nursing are recognized/identified in these contexts, with relative ease by the nursing team, mostly by more experienced nurses, and by family members. Nurses recognize the therapeutic futility of their interventions in the situations mentioned above and when there is a prolonged stay of the person in the intensive care unit without improvement or recovery. It should be noted that one of the participants made a point of mentioning that he cannot associate the identification of pain with the recognition of therapeutic futility because, according to this nurse, currently the teams are highly trained to detect pain and can anticipate potential discomfort situations with relative ease, so these are very infrequent situations. Regarding the family, nurses report that this recognition is, above all, associated with the length of stay and the lack of results. According to them, most family members are perspicacious in identifying these situations and there is an increase in literacy that facilitates the recognition of these situations of futility.

In what concerns to the scope of therapeutic futility in nursing, the nurses’ difficulty in characterizing the dimension and extension of this conception was significant, with the participants showing some reservations, especially in the presentation of proposals for terms/terminology for this dimension. These difficulties seemed to be associated, above all, with the complexity of the topic under study, and not exactly with the inexperience of the participants, who confessed to having experienced numerous situations that they manage to associate with therapeutic futility in nursing.

The analysis of the few national studies carried out with nurses on therapeutic futility made it possible to identify some results that are in line with the findings of this study. These studies, carried out in intensive care and palliative care, which analyze this phenomenon in end-of-life situations, conclude that therapeutic futility is a recurrent event that includes the implementation of excessive interventions that are not associated with benefits, which should be avoided at all costs, for the implications it may have in prolonging suffering and diminishing human dignity (Marinho & Casanova, 2019; Teixeira et al., 2012; Vieira et al., 2021c).

Aiming to reduce the occurrence of therapeutic futility, the authors emphasize, as in most of the available studies, the relevance of carrying out more studies on this phenomenon and the importance of education and training of health professionals on this phenomenon (Domingues et al., 2015; Marinho & Casanova, 2019; Teixeira et al., 2012; Vieira et al., 2021c).

## Strengths and Limitations

The limitations identified for this study are all related to the data collection method, especially with the option of carrying out remotely. Holding an online focus group runs the risk of harming communication, because of the risk of inhibiting participants to participate. Additionally, given the possible stay in a familiar place during the focus group, such as the participants’ own home, where the environment and external stimuli cannot always be controlled, the possibility of some distraction cannot be excluded. Nevertheless, the authors consider that the advantages and benefits of conducting the focus group at a distance outweigh the disadvantages, with particular emphasis on the possibility of including participants from dispersed geographical areas.

## Implications for Practice

This study contributes to the scientific knowledge of nurses in intensive care units about therapeutic futility in nursing and helps these professionals to reflect in advance and systematically about its occurrence in these environments. The development of nurses’ skills in this area is valuable contribution to preventing the occurrence of this phenomenon and all the negative consequences associated with it.

## Conclusions

Therapeutic futility in nursing is a reality perceived by nurses in adult intensive care units and four declared situations are associated with this phenomenon, namely, biophysiological indicators incompatible with life, intensive care culture of life extension, surgical situations of high irreversibility, uncontrollable, associated with severe comorbidities, contexts in which, according to scientific evidence, the results are unattainable and do not justify the implementation of interventions. These situations, which may include futile nursing interventions that fall into four categories, specifically, interdependent interventions, autonomous interventions, complementary diagnostic tests, and care implemented due to scores, can be perceived not only by the nursing team, but also by the family. Four subcategories for the scope of therapeutic nursing interventions are identified, which include ridicule of care, transposing the limits of intervention and care, no benefit, and therapeutic incarceration.
